# The Murri clinic: a comparative retrospective study of an antenatal clinic developed for Aboriginal and Torres Strait Islander women

**DOI:** 10.1186/1471-2393-12-159

**Published:** 2012-12-21

**Authors:** Sue Kildea, Helen Stapleton, Rebecca Murphy, Natalie Billy Low, Kristen Gibbons

**Affiliations:** 1 Australian Catholic University, 1100 Nudgee Road, Banyo, QLD, 4014, Australia; 2 Mater Medical Research Institute, Level 3, Aubigny Place, Raymond Terrace, South Brisbane, Qld, 4101, Australia

**Keywords:** Aboriginal and Torres Strait Islander, Indigenous Australian, Antenatal, Maternity, Midwifery, Culturally responsive, Model of care, Evaluation, Multi-agency

## Abstract

**Background:**

Indigenous Australians are a small, widely dispersed population. Regarding childbearing women and infants, inequities in service delivery and culturally unsafe services contribute to significantly poorer outcomes, with a lack of high-level research to guide service redesign. This paper reports on an Evaluation of a specialist (Murri) antenatal clinic for Australian Aboriginal and Torres Strait Islander women.

**Methods:**

A triangulated mixed method approach generated and analysed data from a range of sources: individual and focus group interviews; surveys; mother and infant audit data; and routinely collected data. A retrospective analysis compared clinical outcomes of women who attended the Murri clinic (n=367) with Indigenous women attending standard care (n=414) provided by the same hospital over the same period. Both services see women of all risk status.

**Results:**

The majority of women attending the Murri clinic reported high levels of satisfaction, specifically with continuity of carer antenatally. However, disappointment with the lack of continuity during labour/birth and postnatally left some women feeling abandoned and uncared for. Compared to Indigenous women attending standard care, those attending the Murri clinic were statistically less likely to be primiparous or partnered, to experience perineal trauma, to have an epidural and to have a baby admitted to the Neonatal Intensive Care Unit, and were more likely to have a non-instrumental vaginal birth. Multivariate analysis found higher normal birth (spontaneous onset of labour, no epidural, non-instrumental vaginal birth without episiotomy) rates amongst women attending the Murri clinic.

**Conclusions:**

Significant benefits were associated with attending the Murri clinic. Recommendations for improvement included ongoing cultural competency training for all hospital staff, reducing duplication of services, improving co-ordination and communication between community and tertiary services, and working in partnership with community-based providers. Combining multi-agency resources to increase continuity of carer, culturally responsive care, and capacity building, including creating opportunities for Indigenous employment, education, and training is desirable, but challenging. Empirical evidence from our Evaluation provided the leverage for a multi-agency agreement to progress this goal within our catchment area.

## Background

Childbearing women and infants constitute a significant proportion of Indigenous Australians, and the impor-tance of prioritising their health has consequences for future generations [1]. Disparities in reproductive health outcomes between Australian Indigenous and non-Indigenous women demonstrate significantly higher maternal and perinatal morbidity and mortality rates amongst the former. For example, maternal mortality is approximately five times greater [2], with a higher proportion of low birth weight infants (liveborn infants: 12.0% vs. 5.6%); preterm births (13.1% vs. 8.0%); and perinatal deaths (18.6 vs. 9.4 per 1,000) [3]. A more unified and comprehensive approach to reducing disadvantage and ‘closing the gap’ in health outcomes between both populations has been proposed [4]. The 2011 report [5] of key maternal and infant health (MIH) indicators shows that despite improvements in some areas (34% decline in perinatal mortality between 1999–2008) anticipated improvements resulting from increased funding through the ‘Close the Gap’ Initiative have not eventuated.

The quality of care delivered by some maternity services is problematic with women expressing dissatisfaction and some reporting racist attitudes [6-9]. One review of the literature in this area claimed many had expressed “*profoundly negative views and experiences of recent and past involvement with mainstream health systems and providers of care including, in particular, hospital clinics”*[10]. Specific issues included: a lack of Aboriginal staff and childcare facilities; poor transport; dissatisfaction with the information provided; long waiting times; negative staff attitudes; poor communication; and limited interpreter services. Care providers identified many of the same issues and the following challenges: cultural gaps between women and themselves; insufficient consultation time; lack of continuity of carer precluding the opportunity for building relationships; and the increasingly complex nature of information sharing required in antenatal care provision (particularly regarding screening tests and their interpretation). Women’s narratives suggested that some viewed consultations with service providers as resembling ‘interrogations’ [11]. The importance, and availability, of choice for younger maternity service users, and their concerns about confidentiality and privacy, have also been identified in a recent report on perceptions of ‘good’ antenatal care [9]. Evaluation results from an urban community-based service, which included interviews with consumers, described a service that made women feel special: *‘For once I was actually treated like it was all about me rather than fitting into everyone else’s schedule’;* and as another woman commented *‘it’s more than just having a baby – it’s about establishing networks, play groups, all sorts of sessions for mums to get together and talk and learn’*[12]*.*

The challenges to effective service delivery are highlighted by population characteristics: Indigenous childbearing women are a widely dispersed population, representing only 3.8% of annual Australian births, and only 27.7% live in major cities compared to 71.6% of non-Indigenous women [3]. Critical reviews of ten antenatal programs found little high quality evidence on which to base recommendations (highest Level III-2) for improving Indigenous MIH due to: wide variation in study design, quality and reporting; small sample sizes; and different treatment effects depending on location [13,14]. Failures regarding inter-agency collaborations were also cited as problematic [13]. Despite these limitations, improvements in antenatal clinic attendance, screening and treatment (e.g. sexually transmitted and urinary infections), immunisation, mean birth weight and rates of preterm birth were noted in some programs [15-18], none of which are universally available in Australia.

Hence, the substantial differences in outcomes remain problematic and highlights the urgent need to develop targeted and innovative programs within embedded evaluation frameworks. Also highlighted in the National Maternity Services Plan is the need to strengthen partnerships between organisations to ensure the provision of clinically safe and culturally competent maternity care [19]. This presents both a challenge and an invitation for clinicians, policy makers and community stakeholders to consider more effective models for delivering MIH services to Indigenous women and their infants, within a comprehensive evaluation framework.

This paper presents results of a recently completed (2010) Evaluation [20] of a specialist antenatal clinic targeting Aboriginal and Torres Strait Islander women: the Murri Clinic (MC). It describes the perspectives of service-users, staff and other stakeholders (internal and external to the hospital). It compares outcomes with Indigenous women who accessed standard care (SC), and considers how maternity care might continue to evolve for the benefit of all Indigenous women and infants. The terms Indigenous and Aboriginal and Torres Strait Islander are used interchangeably throughout this paper.

## Methods

### Aim and objectives

The Evaluation [20] aimed to identify the strengths and challenges of the MC and make recommendations for future development. The objectives were to utilise a participatory approach to undertake a process and impact evaluation by performing a retrospective analysis of maternal and neonatal health data and seeking feedback from service users, staff and other stakeholders.

### Setting

#### Murri clinic

The Murri Antenatal Clinic was established in 2004 in a tertiary Australian hospital where approximately 5,000 women access public maternity services per annum; clinic provision was limited to the antenatal period. Women were referred to the MC if they were identified as Indigenous on the General Practitioner (GP) referral form. Women partnered to Indigenous men also accessed the clinic and women who were identified as Indigenous at a later stage of their pregnancy were offered transfer from mainstream services; clinic staff also accepted self-referrals from eligible women. Clinical input was provided by a hospital-employed Indigenous midwife and a non-Indigenous obstetrician; additional support was available from Indigenous liaison officers (ILOs) who welcomed women and their families and helped them to feel comfortable, and provided support for women transferred in from rural and remote areas. The ILOs also followed up women who ‘failed to attend’ their scheduled appointments; accompanied women to appointments as necessary, and performed an important ‘cultural brokerage’ and advocacy role, particularly for the most vulnerable women. Women requiring the services of Allied Health staff, including social workers, were referred to mainstream services; contracted hours were on a part-time basis for all staff.

#### Standard care

For the purpose of this paper, all other models of antenatal care available at the hospital have been categorised as standard care (SC), including: midwifery and medical clinics (whether community or hospital-based) and specialist services such as Maternal Fetal Medicine. Privately insured women were excluded. Eighteen percent of all women attending the hospital receive care through a Midwifery Group Practice (MGP) which provides continuity across the continuum of the antenatal, birthing and postnatal periods by small teams of midwives, with one acting as primary midwife to the women in her caseload.

Both SC and MC services offer shared care with GPs and the Aboriginal Medical Services (AMSs) located within the hospital catchment area.

#### Ethics

The Evaluation proceeded following approval from the hospital Human Research Ethics Committee.

### Study design

A triangulation mixed method approach [21] was used to generate qualitative and quantitative data from a range of sources (individual and focus group interviews; surveys; mother and infant audit data; and routinely collected data (from hospital databases) gathered concurrently over a 12-month period. To inform future service development we mapped the postcodes of service users’ places of residence against the 2006 census (suburb and postcode) using Geographic Information System data to identify the localities of women accessing the MC. The data sets were combined in the intermediate stage of the Evaluation for interpretation and analysis, which highlighted issues necessitating further data collection (for example regarding shared care), including a chart audit of 2009 MC clients.

Two female peer research assistants (one Aboriginal and one Torres Strait Islander) received research training and were employed part-time for recruitment and data collection; one continued her employment and participated as an author of this paper. A reference group, comprising Indigenous and non-Indigenous personnel including MC staff and representatives from allied health and hospital management, met fortnightly throughout the Evaluation. They helped the research team troubleshoot problems, provided support and guidance regarding culturally sensitive issues, and gave feedback on the draft report. The researchers met regularly with staff from the AMSs throughout the Evaluation; staff from one AMS helped design a survey and staff from both assisted with recruitment and promotion of the Evaluation within their organisation.

### Participants

Research participants included MC staff and service users; hospital managers and staff; and relevant community stakeholders, and representatives from a variety of community organisations including two AMSs. The qualitative data collection period occurred from March-October 2010. Although the sample of Indigenous service users was smaller than anticipated our data were varied and included the perspectives of young women (both Aboriginal and Torres Strait Islanders), multiparous and primiparous women, women partnered to Aboriginal men, and older women.

### Recruitment and data collection

#### Service users

Recruitment of this group was challenging as privacy legislation prevented the MC staff, and those in community-based facilities, from providing women’s contact details. The Evaluation teamwere thus heavily reliant on staff to promote the Evaluation directly to women and to disseminate fliers and other information materials through their community networks. Women who indicated an interest, and who consented to their contact details being passed onto the Evaluation team, were then contacted by one of the peer interviewers who arranged to meet them at a mutually convenient time and place. Some participants were identified by snowball sampling whereby women who had participated in the Evaluation recruited friends and relatives. Women were offered the opportunity to complete one or two paper based surveys; the ‘Yarning Circles’ survey was developed in consultation with key stakeholders from one AMS and focused on antenatal education; a more general survey focused on women’s experiences of using the MC. Both surveys contained ‘free text’ options for women to write additional comments; the peer interviewers helped women with literacy problems to complete surveys, sometimes acting as ‘scribes’ to dictate their feedback. Women were also offered 1-to-1 semi-structured interview or participation in a focus group. Consent was implied for women completing surveys; written consent was obtained from those who contributed interviews.

#### Staff

Hospital staff in relevant clinical areas were emailed and invited to complete a web-based survey which sought demographic data and information about a range of issues including staff confidence and experiences of working with Indigenous women/families, and cultural awareness training. Reference group members actively championed participation and two reminder emails were dispatched over a six-week period. A paper copy of the survey was also distributed throughout key areas. Other staff, including managers and those with specific responsibilities for the MC, were invited to contribute individual, or focus group, interviews (none declined).

#### External stakeholders

The reference group recommended external stakeholders, including staff from both AMSs; all who were approached agreed to be interviewed.

Interviews and focus groups were arranged at mutually convenient times and places; written consent was obtained prior to participation. Refreshments were provided and travel and childcare costs were reimbursed. All interviews, which were in English, were audio recorded and transcribed verbatim.

### Data sources

**Figure 1:**
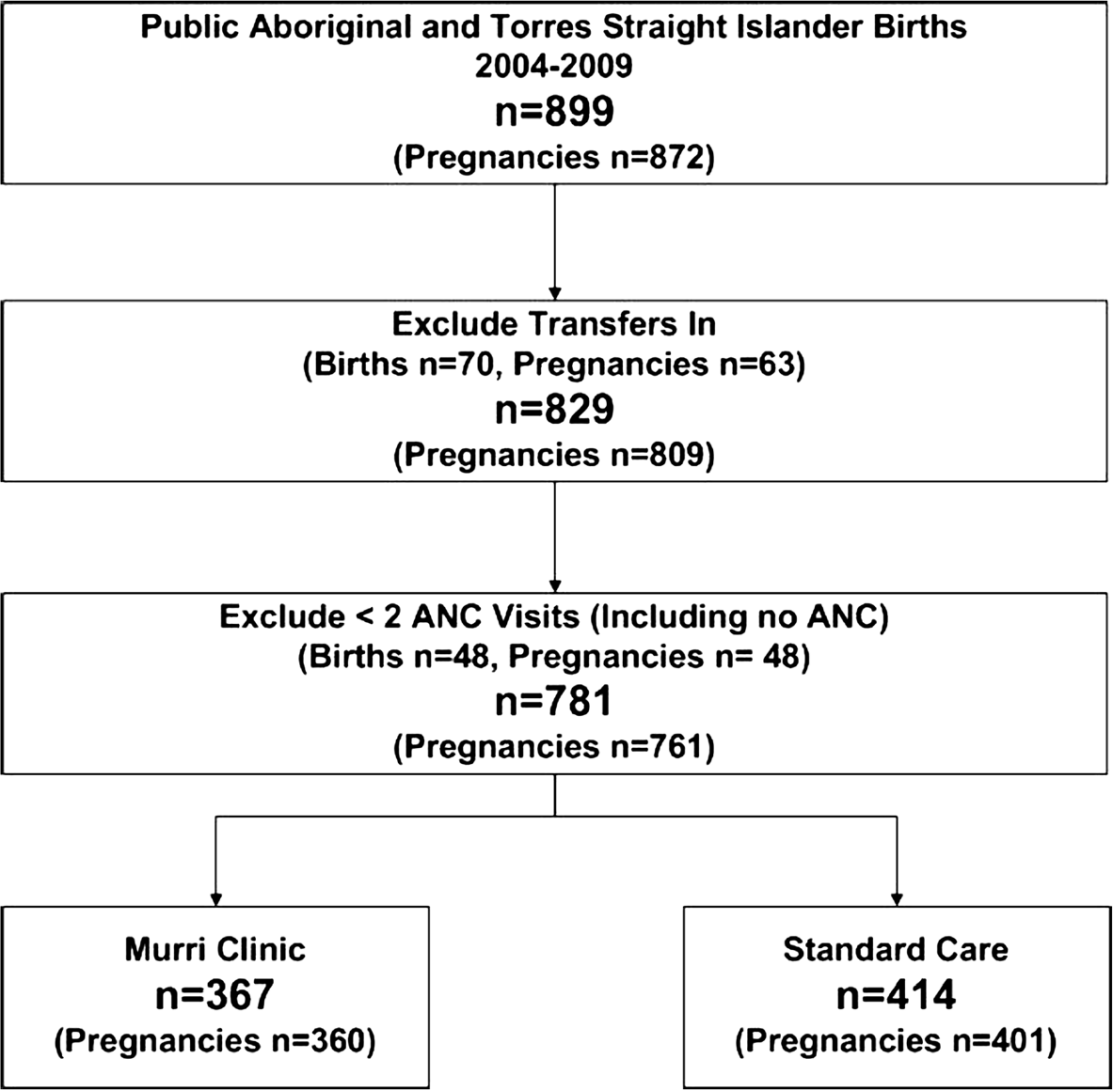
Flowchart of included participants.

A total of 220 participants contributed data: 46 service users (38 completed surveys with an additional eight contributing 1-to-1 interview data); 157 staff (147 completed surveys [21% response rate], with an additional ten interviewed 1-to-1 or in pairs); 17 external stakeholders contributed data through focus groups or 1-to-1 interviews. Routinely collected clinical and psychosocial quantitative data were obtained from two obstetric databases (Obstetric Clinical Reporting System, Clinical Reporting Systems Pty Ltd, New South Wales (NSW), Australia and MatriX, Meridian Health Informatics, NSW, Australia) for 367 women (the MC cohort) who attended the clinic between 2004–2009, and the 414 Indigenous women attending SC services in the same hospital over the same time period (Figure 1). A chart audit was conducted on a random sample of 50% of 2009 service users (n=59) in order to understand more about the shared care (between hospital clinicians, GPs and the AMSs) arrangements for these women. Some variables were only available for May 2007–2009. All percentages presented are calculated on non-missing data with the number available for analysis detailed in tables below.

### Data analysis

Clinical data were collated and analysed using StataSE Version 10 (StataCorp, College Station, Texas, United States of America). Survey data were collated and analysed using SPSS Version 17 (IBM Corporation, Chicago, United States of America). Graphical interpretations of quantitative results were generated using Microsoft Office Excel 2002 (Microsoft Corporation, Redmond, Washington, USA). Bivariate analysis comparing outcomes between the two cohorts, MC attendees (n=367) and SC attendees (n=414), are reported as the difference in proportions with associated 95% confidence intervals (CIs). Statistical significance was set at the 0.05 level. Multivariate logistic regression was undertaken adjusting for the following possible confounders: age, body mass index (BMI), smoking status, specific pregnancy complications (gestational diabetes, antepartum haemorrhage and/or pregnancy-induced hypertension) and socio-economic status as measured by Socio-Economic Indexes for Areas (SEIFA) [22]. Results are reported as adjusted odds ratios (aOR) and associated 95% CIs. Due to small sample sizes for both staff and participant surveys no statistical inference was undertaken for these data. Results reported in tables are not repeated in the text.

A sample of interview transcripts was read by two of the authors [B & C] who identified key themes and indepen-dently created a coding system. Codes were compared, inconsistencies discussed and reconciled, and a final coding scheme agreed. To reduce the likelihood of errors, precise descriptors were written for each code. Coded transcripts were entered into NVivo Version 8 (QSR International Pty Ltd, Doncaster, Victoria, Australia), key themes identified, and thematic analysis undertaken [23,24].

## Results

### Maternal characteristics

Maternal age and BMI were similar across the two cohorts (Table 1). Compared to Indigenous women attending SC, MC women were less likely to be partnered or primiparous, and more likely to be multiparous (greater than 3 births). The proportion of MC women with iron deficiency anaemia was almost half. There were no statistically or clinically significant differences between the two cohorts for any of the other conditions analysed including pregnancy induced hypertension, gestational diabetes and antepartum haemorrhage (data not shown). The rates of key psychosocial indicators were similar across cohorts. Some clinically significant differences were noted in the Murri cohort in terms of alcohol consumption during pregnancy, smoking at first hospital (booking) visit and Department of Child Safety (DOCS) involvement; however no statistically significant differences were found.

**Table 1:** Maternal characteristics and key social indicators by antenatal model of care, 2004-2009

**Indicator**	**Definition**	**MC**	**SC**	**% difference**	**p**
		**n (%)**	**n (%)**		
Age	<20	61 (16.9)	73 (18.2)	−1.3	0.587
	20-24	96 (26.7)	106 (26.4)	0.3	
	25-29	80 (22.2)	106 (26.4)	−4.2	
	30-34	74 (20.6)	75 (18.7)	1.9	
	35-39	40 (11.1)	33 (8.2)	2.9	
	40+	9 (2.5)	8 (2.0)	0.5	
BMI	Underweight (≤18.50)	26 (8.8)	32 (9.3)	−0.6	0.207
	Optimal (18.51-<25)	121 (40.9)	164 (47.8)	−6.9	
	Overweight (25–30)	71 (24.0)	78 (22.7)	1.3	
	Obese (>30)	78 (26.4)	69 (20.1)	6.2	
Parity	Primiparous	103 (28.7)	151 (38.2)	−9.5	0.002
	One	82 (22.8)	99 (25.1)	−2.2	
	Two	55 (15.3)	63 (16.0)	−0.6	
	Three	45 (12.5)	36 (9.1)	3.4	
	More than three	74 (20.6)	46 (11.7)	9.0	
Marital status	Married/de facto	153 (46.5)	240 (62.2)	−15.7	<0.001
	Single/never married	165 (51.6)	126 (35.2)	15.8	
	Divorced/separated	8 (2.4)	10 (2.6)	−0.2	
Cannabis use at first visit	Asked at booking	34 (9.5)	24 (6.0)	3.5	0.071
Alcohol consumption during pregnancy	Asked at booking	22 (6.1)	33 (8.2)	−2.1	0.260
Smoking	Asked at booking	188 (52.4)	184 (46.1)	6.3	0.086
Iron deficiency anaemia ^a^	Asked at booking pre-existing or current issue	13 (7.1)	22 (14.7)	−7.5	0.026
Domestic Violence (afraid for physical safety) ^a^	Asked at booking	5 (3.0)	4 (3.3)	−0.3	1.000
Domestic Violence (emotional abuse) ^a^	Asked at booking	8 (4.9)	8 (6.7)	−1.8	0.605
DOCS involvement ^a^	Asked at booking “Have you ever had any help from DOCs”	14 (13.9)	15 (10.0)	3.9	0.287

^a^ 2007–2009 only.

High rates of smoking were evident in both cohorts; 81% of survey participants stated they had received information on smoking in pregnancy, although women not infrequently complained that:

"I don't want to be told not to smoke over and over again. (Participant survey)"

### Service provision

#### Accessing the clinic

Difficulties with internal referral pathways, and identification of Indigenous status, resulted in some women who were eligible to access the MC, being allocated to mainstream services. This was sometimes rectified after women heard about the clinic and negotiated a transfer. Significant delays were identified concerning referral processes, reiterated by interview data, with the average referral for MC women received at 16 weeks gestation (range 5–37 weeks) with a further eight weeks wait on average to attend a ‘booking’ visit which occurred at 24 weeks vs. 20 weeks (mean) for Indigenous women in SC. Indigenous women attending SC were statistically more likely to attend eight or more visits (51.0% vs. 38.4%), although the hand held record which we accessed for audit purposes, is usually only commenced at the first hospital booking visit with previous episodes of community based care rarely recorded. Hence, antenatal attendance for women accessing MC shared care is likely to be under-recorded.

#### Shared care

Women accessed the MC from a wide geographical region including outside the hospital catchment area. Routinely collected data provided limited information on GP shared care arrangements (MC:14% vs. SC:34%) with interview data suggesting the proportion of MC women receiving shared care was higher. We instigated a chart audit of 2009 data and identified a much higher proportion (68% vs. 14%) participated in shared care with a variety of providers including two AMSs (39% and 15%); and GPs (MC: 14%). Participants expressed a desire for greater collaboration and partnership across organizations, believing that a team approach would be more effective and efficient, and reduce the substantial amount of photocopying and faxing of patient records between services:

"Everyone’s got their own patch. […] Everyone seems to be doing something but nothing. (Staff)"

"They’ll drop in every three weeks […] even though they don’t need antenatal care […] you know there is kind of a doubling up. (External stakeholder)"

Women mentioned needing to tell their story numerous times to different providers:

"All they do is ask you the same questions. (Participant)"

Interview data highlighted concerns from hospital staff and external stakeholders about the lack of co-ordinated care and duplication of services, for example due to different pathology providers, screening test results which were inaccessible for ‘after-hours’ admissions.

#### Continuity of carer

At the time the Evaluation was undertaken the MC operated one day a week; midwifery appointments were available all day with an obstetric clinic operating alongside in the afternoon. Continuity of carer (midwife and obstetrician) was a major attraction, with women reporting that this reduced the likelihood of being asked the same questions:

"It’s good coming here too because you know you’re going to see the same people all the time. It’s not a different doctor or a different midwife every time who’s going to ask you the same questions over and over again […] she (midwife) knows your full-on history from the first visit to, you know, your last visit. She knows everything about you, which is good. (Participant)"

Survey data confirmed that continuity of midwifery carer was very important to women attending the MC, with continuity of obstetrician and social worker slightly less so (61% vs. 47% vs. 42%). Clinic staff believed that the social worker provision, with minimal or no continuity, discouraged women’s frank and honest disclosures about their troubles. For their part, Social Workers reported being disappointed when their efforts to engage ‘hard to reach’ women were thwarted because they were unable to secure the level of continuity required to ensure culturally safe practice.

Staff were further concerned that lack of continuity in this area jeopardised the clinical management strategies in place for ‘high risk’ women; they were also concerned that this might be a factor in such women disengaging from all care provision. Although staff considered Indigeneity to be an important feature of the MC, their clients seemed less concerned about the Indigenous status of staff, stipulating that more important was access to the same care provider who was well qualified and experienced, with good listening skills:

"I’m more concerned about their qualifications and how much experience they’ve had […]. I’m not really worried about whether they’re Indigenous or not. (Participant)"

"It’s someone that’s going to listen to you, then it don’t matter what they look like. (Participant)"

#### Operational issues

The limited clinic opening hours were considered insufficient to meet demand. Lack of flexibility with schedu-ling appointments was a particular problem which staff believed deterred some women from booking with the MC.

"Sometimes they don’t come to this clinic because […] we’re only on a Thursday and it doesn’t suit everyone […] maybe the family’s working […] studying, and they can’t get here on a Thursday and for that reason they’ll go through mainstream. (Staff)"

Limited provision increased pressures on staff as the clinic was extremely busy on the one day it was in operation; women reported that their appointments rarely ran to time. Staff agreed that delays were frequent and often substantial, commenting that scheduled appointments were also not in keeping with Indigenous cul-tural norms, which favoured ‘drop in’ arrangements. An important concern for staff was that if they did not accommodate women who dropped in to the clinic unannounced, and who were more likely to be at higher risk with poor attendance records, a crucial opportunity for antenatal care would be lost. At the risk of inconveniencing women who were punctual with appointments, staff adopted an opportunistic approach to these less regular clients:

"Like she had an appointment two hours ago and now she’s here and it’s like so, I’ll just see her […] Indigenous health as you know is opportunistic and if you don’t get them then, you might not get them again. (Staff)"

Women sometimes used the knowledge that they would be seen without an appointment to their advantage:

"I figured out how it (the clinic appointment system) was working. So I would go up there like (at) nine (am) […] women would be waiting after me that were probably meant to be before me. (I felt) bad at first but, you know, that’s the way they operate. (Participant)"

Strategies which assist patients to circumvent perceived organisational deficits may be important for survival in systems which are not designed to take account of their individual needs. The efforts expended by staff to balance opposing needs and professional responsibilities, however, are also acknowledged as considerable [25,26].

#### Location

Clinic staff and external stakeholders were of the opinion that the location of the clinic, within a tertiary maternity hospital setting, was problematic with some believing this hindered women’s attendance at clinic appointments. In particular, they cited long journeys, with perhaps multiple changes on public transport, travel-related costs including parking fees, and problems associated with travelling accompanied by small children and pushchairs. Women who contributed to the Evaluation, however, refuted these suggestions, stating that accessing the clinic was relatively easy. Women who drove to the clinic for their appointments reported that they frequently parked in side streets where parking was cheaper (or free) rather than using the (expensive) hospital car parks. Busways (i.e. dedicated roadways separating buses from general traffic), were a popular alternative for those travelling by public transport, not least because the bus stop was located directly in front of the hospital. When women were asked their preferences regarding the location of the clinic, some suggested community-based locations, closer to where they lived. However, the hospital was also favoured for the opportunity it provided partners and family members to become familiar with the surroundings in advance of labour and admission to birth suite:

"I could be in labour and he’d have to bring me here and not know where to go. (Participant)"

#### Physical space

Complaints about overcrowding and lack of privacy to discuss confidential matters were frequently articulated. One participant referred to the waiting area as a 'fishbowl’:

"It’s a very fishbowl kind of situation. We’re all in there looking at each other in a very small space and as soon as the woman comes in with her other two kids and they start playing in the middle of the space, we’re all part of it you know? It’s like the comfort zones might be pushed for some […] I think it definitely needs a better situation […] at least a more appropriate one. (Participant)"

Male partners in particular were reported as being loathe to spend time in the waiting room, opting instead to use mainstream facilities:

"Yeah they (male partners) wait outside […] (they) don’t feel comfortable. Like my partner didn’t feel comfortable sitting in there […] sometimes I’d see just fathers just stroll past […] because they don’t really want to go in there. (Participant)"

In addition to concerns about confidentiality, staff believed lack of space hindered their ability to properly engage with women and their families. This was problematic as building rapport and establishing trusting relationships was an essential prelude for staff attempting to address the complex, and often long-standing, health-related risk factors disrupting the lives of so many of their clients.

#### Culturally responsive care

Analysis of survey data found that the majority of women (92%) felt ‘mostly understood and respected’ by staff whilst attending the MC. Other hospital locations were less well rated, however, with only 47% of women feeling similarly about birth suite, 31% about the postnatal ward and the 31% about the Maternal Fetal Medicine unit. Relatively small percentages of women stated they felt ‘not at all understood’ or ‘respected’ in selected locations including; birth suite (14%), the Neonatal Intensive Care Unit (NICU) (6%), the postnatal ward (8%), the Maternal Fetal Medicine unit (8%), and the Home Care Program (6%). Women described their disappointment with the lack of continuity of carer they experienced in birth suite and postnatally. Being cared for by unfamiliar staff during labour was especially difficult for younger Indigenous women, some of whom reported feeling ‘scared’ and/or ‘shamed’ when staff they had not previously met entered their room unannounced. Their discomfort was heightened by intrusions that occurred when clinical assessments were taking place, with women of the opinion that the intimate nature of labour and birth called for a known carer and that all other staff should seek permission to attend:

"I was kind of like covering myself. (I felt) shame. […] was like, embarrassed. (Participant)"

"I think it’s a very private time labour, and I think you should have a choice as to who is to be there. (Participant)"

Staff generated anxieties about labour progress and infant welfare when they failed to discuss with women in their care the need for consultation with medical colleagues:

"It makes you panic more when more people walk in because it makes you think that there’s something wrong […] staff walk in and you think, what the hell is going on? Something’s going wrong here, is it going to be all right? (Participant)"

### Clinical outcomes

When compared to Indigenous women who attended SC, women who attended the MC were statistically less likely to experience perineal trauma, undergo an elective caesa-rean section, and have a baby admitted to the NICU (Table 2). Women attending the MC attended less ANC visits at the hospital, however we did not have accurate records of the number of visits with other providers.

**Table 2:** Key maternal and neonatal indicators by antenatal model of care, 2004–2009

**Indicator**	**Definition**	**MC**	**SC**	**% difference**	**p**
		**n (%)**	**n (%)**		
Number of antenatal visits at MC or hospital	2-4	57 (15.8)	44 (11.0)	4.9	0.007
	5-7	85 (23.6)	79 (19.7)	3.91	
	≥8	101 (28.1)	151 (37.7)	−9.5	
Preterm (<37 weeks)		44 (12.2)	58 (14.5)	−2.2	0.365
Onset of labour	Spontaneous	245 (68.1)	261 (65.1)	3.0	0.101
	Induced	81 (22.5)	82 (20.5)	2.1	
	No labour- c/s performed	34 (9.4)	58 (14.5)	−5.0	
Mode of birth	Non-instrumental vaginal	254 (70.6)	250 (62.5)	8.1	0.060
	Forceps	5 (1.4)	3 (0.8)	0.6	
	Vacuum	15 (4.2)	26 (6.5)	−2.3	
	Caesarean section	86 (23.9)	121 (30.3)	−6.4	
Analgesia	Inhalational	167 (46.8)	183 (47.0)	−0.3	0.942
	Opioid	98 (27.5)	99 (25.5)	2.0	0.536
	Epidural	68 (19.1)	101 (26.0)	−6.9	0.024
5 min Apgar <7 ^a^		4 (1.1)	9 (2.2)	−1.1	0.274
Perineal trauma ^b^	Intact or 1^st^ degree tear	263 (96.0)	230 (83.2)	12.8	<0.001
	2^nd^ degree tear	7 (2.6)	32 (11.5)	−8.9	
	3^rd^ or 4^th^ degree tear	0	2 (0.7)	−0.7	
	Episiotomy	4 (1.5)	13 (4.7)	−3.2	
Low birth weight (<2500g)		46 (12.5)	60 (14.5)	−2.0	0.425
Post partum haemorrhage (>500mls blood loss)		44 (12.3)	46 (11.5)	0.8	0.738
NICU admission ^a^		58 (15.9)	90 (21.8)	−5.9	0.036
Feeding at discharge ^a^	Breast milk	234 (69.4)	262 (72.2)	−2.7	0.248
	Breast & formula	27 (8.0)	22 (6.1)	2.0	
	Formula	73 (21.7)	70 (19.3)	2.4	
	Gavage	3 (0.9)	9 (2.5)	−1.6	

^a^ Excluding stillbirths ^b^ Excluding Caesarean sections.

Multivariate analysis found that Indigenous women who attended the MC were significantly more likely to have a normal birth with no statistically significant difference seen for preterm birth or admission to the NICU (Table 3).

**Table 3:** MC vs. SC bivariate and multivariate analysis of key clinical outcome indicators

**Outcome**		**Bivariate**			**Multivariate**		
	**N**	**Odds Ratio**	**95% CI**	**P**	**Odds Ratio**	**95% CI P**	
Preterm <37 weeks ^a^	648	0.92	0.59, 1.43	0.710	0.92	0.58, 1.46	0.732
Normal birth ^a,b^	648	1.61	1.17, 2.20	0.003*	1.48	1.07, 2.06	0.019*
NICU admission ^a,c^	643	0.71	0.47, 1.06	0.100	0.70	0.45, 1.08	0.110

^a^ Adjusted for maternal age, parity, body mass index, smoking, medical complications (gestational diabetes, antepartum haemorrhage, pregnancy induced hypertension, asthma) and socio-economic status as measured by the Socio-Economic Indexes of Areas.

^b^ Normal birth defined as: spontaneous onset of labour, no epidural, non-instrumental vaginal birth and no episiotomy.

^c^ Excluding stillbirths *CI* Confidence Interval. * Statistically significant.

## Discussion

Culturally appropriate and safe care is paramount to maternity provision for all childbearing women and their families; a well-established literature [27,28] confirms that health-related interventions are more likely to succeed when tailored to fit the socio-cultural context. This is particularly true for Indigenous Australians whose understandings of concepts such as ‘health’ and ‘well-being’ may differ markedly from Western bio-medical approaches [29]. Common factors associated with successful and culturally appropriate services for Indigenous mothers and babies have been identified and include, but are not limited to: community-based and/or community controlled services, a dedicated and specifically designed venue, continuity of carer, and a broad spectrum of well-integrated allied health and other specialist services [13,14]. Our Evaluation confirmed that continuity of antenatal carer, additional support from the hospital-based Indigenous liaison team, and culturally sensitive practices (e.g. being aware of cultural norms and family responsibilities, and communicating appropriately), were successful elements in the model of care provided by the MC. That said, little detail was provided about the constituent elements of a culturally sensitive service. Women consistently reported that these aspects of service provision protected them from having to repeat information and afforded opportunities to build trusting relationships.

The majority of women who participated in the Evaluation reported that they were happy with the clinic location and care arrangements (lack of continuity notwithstanding); however, dissatisfaction may have been a factor motivating Indigenous women who attended other facilities or selected SC (53.2% of all Indigenous women attending the hospital). We were unable to ascertain if these women were ever offered the MC, or if they declined this option. The authors acknowledge that lack of data in this respect is a weakness in the study findings.

Our findings strongly suggested that employment of an Indigenous midwife and ILOs was very well received by both women attending the MC and other care providers, whether hospital or community-based. In particular, hospital staff believed the contribution made by their Indigenous colleagues helped to ensure a culturally safe and appropriate environment. Additionally, women emphasised the importance of staff qualifications and expertise, continuity, and communication skills.

Deficiencies in staff communication and interpersonal skills were particularly noticeable when women transitioned from antenatal to labour and postnatal environments where care was provided by unfamiliar staff. Previous research confirms that Indigenous women encounter culturally inappropriate maternity services and discriminatory attitudes amongst staff [30] although this was not a significant feature in our research. That said, some women did confide that they felt staff did not always listen to them and sometimes discounted their opinions and version of events. Some also described occasions where their privacy was intruded upon, for example during clinical assessments in labour; this heightened their sense of shame and embarrassment.

Our findings suggest a positive association between the care provided to women attending the MC and some outcomes e.g. reduced perineal trauma, epidural administration and NICU admission, and higher rates of non-instrumental vaginal birth and normal birth (spontaneous onset of labour, no epidural, non-instrumental vaginal birth without episiotomy). After adjusting for known confounders we still found a positive and statistically significantly difference in normal birth rates, and a trend towards reduced NICU admissions. These may be due to women with higher medical risk factors (not found in our data), attending SC although we cannot rule out a possible association with the care provided through the MC. Psychosocial and lifestyle data reflected high rates of complexities in this population of women. This may help to explain the willingness of MC staff to spontaneously extend antenatal visits and ‘slip in’ women for consultation ahead of others already waiting, sometimes considerably beyond their appointment time.

Our findings confirmed that duplication of services between the MC and external services (such as AMSs) is problematic. The fragmented nature of shared care and suboptimal communication between hospital and community-based providers contributed to operational inefficiencies. Despite the efforts of all concerned, the current model of care offered by the MC cannot be described as woman-centered. In the absence of standardised protocols and reliable systems for information sharing, multi-agency maternity provision is not ideal and indeed, may impact negatively on the quality of care provided. However, due to the participatory nature of the Evaluation, changes to service provision intended to decrease delays in referral processes, and inaccurate or incomplete recording of data (e.g. BMI, smoking), have commenced.

A common, and widely acknowledged, successful feature of Indigenous-specific services is that they are either community-controlled or work within a community model. It is yet to be determined however whether, and how, it might be possible to streamline local provision to provide a ‘gold class’ maternity service across all sectors, including primary and tertiary, non-government and State. Models of care such as midwifery group practices, which provide a high degree of continuity throughout pregnancy, birth and the postnatal period, and are known to positively influence MIH outcomes [31-33], are being introduced in some Australian settings with a variety of Indigenous workers embedded in the models. Early evaluations of these models show promising results [34-36].

Clinical privileging, or access rights, are required if midwives are to be employed in primary care settings and offer continuity during birth (at the tertiary centre) and beyond. Currently, however, such arrangements are in their infancy in Australia. Combining multi-agency resources to increase continuity of carer, including during birth, culturally responsive care, Indigenous employment, capacity building, education and training is desirable, but has rarely been achieved in the area of maternity service provision. Finally, services that provide on-going cultural competency training as a matter of routine would go some way to improving staff knowledge and skills, and hence work towards reducing iniquitous care to a population with some of the poorest MIH outcomes.

### Limitations

Small numbers, and the retrospective nature of the Evaluation, limit the conclusions that can be drawn from the data. Having access to a contemporary comparison group however, greatly strengthened our findings not least because there is little existing empirical research in this national priority area.

## Conclusions

The Evaluation results and recommendations have been presented to, and endorsed by, the hospital leadership team. Dissemination has included presentations to hospital and AMS staff, and an overview of results has been sent to other stakeholders. High-level discussions have been held with several AMSs and agreements to move towards a multi-agency approach, and to jointly apply for research funding, have been made. A World Café style workshop was recently held with 60 participants (including community elders, service users, external and internal stakeholders) to present the results, discuss recommendations and solutions, and design a new model of care.

## Competing interests

There are no competing interests, financial or otherwise, for any of the authors.

## Authors’ contributions

Author contributions are as follows: SK conceived of and led the study, and participated in the design, conduct and coordination. SK also helped draft the manuscript. HS assisted in the coordination of the project, led data collection and qualitative analysis of data. HS also helped draft the manuscript. RM helped in the qualitative analysis of data, conducted the quantitative analysis and helped draft the manuscript. NL collected data and helped draft the manuscript. KG oversaw the quantitative analysis and helped draft the manuscript. All authors read and approved the final manuscript.

## Pre-publication history

The pre-publication history for this paper can be accessed here:

http://www.biomedcentral.com/1471-2393/12/159/prepub
